# Chemical Modifications of Continuous Aramid Fiber for Wood Flour/High-Density-Polyethylene Composites with Improved Interfacial Bonding

**DOI:** 10.3390/polym13020236

**Published:** 2021-01-12

**Authors:** Wanyu Liu, Yue Li, Shunmin Yi, Limin Wang, Haigang Wang, Jingfa Zhang

**Affiliations:** 1 Key Laboratory of Bio-Based Material Science and Technology (Ministry of Education), Northeast Forestry University, Harbin 150040, China; liuwanyu11@163.com (W.L.); wlm8521@163.com (L.W.); 2 Taiyuan Science Institute of Soil and Water Conservation, Taiyuan 030000, China; liyue931020@163.com; 3 Guangxi Key Laboray Chemistry & Engineering Forestry Production, Guangxi University Nationalities, Nanning 530008, China; shunminyi10@163.com; 4 Forest Research Institute, Université du Québec en Abitibi-Témiscamingue, Rouyn-Noranda, QC J9X 5E4, Canada

**Keywords:** continuous aramid fiber, chemical modification, interfacial strength, mechanical properties

## Abstract

To expand the use of wood plastic composites in the structural and engineering constructions applications, continuous aramid fiber (CAF) with nondestructive modification was incorporated as reinforcement material into wood-flour and high-density-polyethylene composites (WPC) by extrusion method with a special die. CAF was treated with dopamine (DPA), vinyl triethoxysilane (VTES), and DPA/VTES, respectively. The effects of these modifications on compatibility between CAF and WPCs and the properties of the resulting composites were explored. The results showed that compared with the original CAF, the adhesion strength of DPA and VTES combined modified CAF and WPCs increased by 143%. Meanwhile, compared with pure WPCs, CAF after modification increased the tensile strength, tensile modulus, and impact strength of the resulting composites by 198, 92, and 283%, respectively.

## 1. Introduction

With the urgent concerns of global warming and depletion of petroleum raw materials, the development of environmentally friendly construction materials has received widespread attention. Wood-polymer composites (WPC) are economic and environmental-friendly materials that have been widely utilized in industry and production (such as the building and automotive industries) due to the recyclability, low costs, dimensional stability, and other advantages of these materials [1,2]. However, plastic and wood fibers are incompatible in WPCs due to their polarity difference, which reduces the strengths and toughness of WPCs [3,4]. For this reason, compatibilizers such as maleated polyolefins [5,6,7], isocyanates [8], and silanes [9] are commonly added to improve interfacial compatibility and partially offset the negative effect of weak interfacial bonding of the composites. However, even with the addition of these compatibilizers, the performance of the WPCs with improved interfacial is still insufficient. In particular, its low impact resistance fails to meet the simultaneous requirements of stiffness and impact resistance for structural applications. Therefore, new strategies for improving the mechanical properties of WPCs are needed.

Hybridization of wood flour with artificial fibers (such as carbon, glass, and aramid fibers) was commonly used to enhance the mechanical strength and impact resistance of WPCs [10]. The addition of short fibers at an appropriate content can effectively improve the performance of the resulting composites, and the properties are related to the fiber types and content [11,12]. Hybrid waste sisal fibers and carbon and glass fiber reinforced polypropylene (PP) composites showed great mechanical and tribological performance, while the properties of composites hybrid with glass fibers were better than carbon fibers for the same hybrid ratios [13]. Commonly, glass fiber has been extensively adopted to reinforced WPCs [14,15]. The strength, modulus of elasticity, and especially the hardness of WPCs increased further by hybridizing small amounts of glass fibers [15,16]. However, adding glass fiber increases the density of resulting composites and causes severe abrasion to machines during processing [13]. Compared with glass fibers, high-performance aramid fiber (AF) possesses a low density and can be used as an ideal reinforcement material in WPCs composites [17]. Adding a small amount of aramid fiber (2–5 wt%) can significantly improve the mechanical properties of WPCs when there was a good interface [18]. However, the surface of aramid fibers is inert and has poor compatibility with most matrix. To improve interfacial compatibility between aramid fibers and WPCs matrix, the fiber has been modified by chemical treatment [18,19] and fiber micro fibrillation [17]. However, even with these modification strategies, the performance of short fiber reinforced WPCs composites remains limited that significantly reduces the potential application of short fiber as reinforcement in WPCs. This problem cannot be addressed by the addition of higher amounts of fibers, as the properties of the finally fiber reinforced composites start to decrease due to fiber agglomeration during processing [11]. Thus, there are limitations of short fiber reinforced composites that remain to be addressed.

Replacing short fibers with continuous fiber into WPCs can solve the problem of fiber agglomeration. Moreover, continuous fiber can withstand a greater load than short fiber and is less easily broken. Compared to short fiber, the use of continuous fiber in WPCs more obviously enhances the mechanical properties [20]. However, due to the complicated preparation process, there have few literature reports on continuous fiber reinforced WPCs. Tamrakar et al. bond a reinforcing sheet of glass fiber and polypropylene (PP) onto the surface of WPC panels using a double-belt pressing method, and observed significantly improved mechanical performance [21]. However, hot-pressing is a complicated, semi-continuous processing method, limiting its application [22]. As an alternative, the extrusion manufacturing process has been applied to fabricate continuous fiber reinforced composites due to simplicity [23,24,25,26]. To do this, a specially designed die is often used to embed continuous fibers into the extruded WPCs.

The performance of continuous fiber-reinforced WPCs composites is dependent on the fiber type, fiber amount, and position of fiber in composites. Whether fiber is embedded into the WPCs or applied to the surface results in different effects on properties of the final composites [27]. In Zolfaghari’s study, 6 rovings of continuous glass fiber incorporated into WPCs increased impact, tensile strength, and flexural strength 20-, 5.9-, and 2.3-fold, respectively [25]. Effects of the amount and type of the continuous fibers on tensile, flexural, and impact strength of WPCs are investigated in our previous work, the impact strength of carbon fiber reinforced WPCs composites improved by 713.4% [26]. Besides, it found that glass fiber has the best interfacial bonding among the three different fiber types (aramid, carbon, and glass fibers). Carbon and aramid continuous fibers would be pulled-out from composites when it is broken due to the weak adhesion between fiber and WPCs. In fact, the properties and life expectancy of continuous fiber-reinforced WPCs are significantly affected by the strength and stability of their interfaces in actual application. The poor interfacial strength between continuous fiber and WPCs matrix significantly reduces the reinforcing effect of continuous fibers. On the contrary, improved properties of continuous fiber-reinforced WPCs composites were observed with improving interfacial compatibility between aramid fibers and the WPCs [18], required for the use of WPCs for construction applications.

Inspired by the mussel, dopamine (DPA) was found can deposit a thin polymer film on almost any substrate through oxidative and self-polymerization [28]. DPA polymer is readily constructed on most substrates without any complex chemical method, so also used as a versatile and effective surface modifier for synthetic fibers (carbon, glass, and aramid fibers) [29,30,31]. Additionally, the deposited poly (dopamine) layer contains many –OH and –NH groups, which can be used for further functionalization [32,33]. Modification by DPA has been proved for improving the compatibility and performance of fiber reinforced composites [29,34,35]. Meta-aramid (MPIA) fiber was first modified by dopamine and further grafted silane KH560 by Sa and the interfacial strength between modified aramid fibers with rubber matrix increased by 62.5% [32]. Grafted polydopamine on carbon fiber can introduce functionalization groups without breaking fiber strength and increased the interfacial adhesion strength and impact properties of the final composites by 78.57 and 75.12%, respectively [35].

Effects of nondestructive fiber modification on the interfacial strength between continuous aramid fiber (CAF) and WPCs and the properties of the resulting composites were explored in this study to expand the application of WPCs in the construction materials. Specifically, the experiment investigated the effect of nondestructive surface modified CAF with dopamine (DPA) and vinyltriethoxysilane (VTES) on interfacial shear strength (IFSS) and mechanical properties of continuous aramid fiber reinforced wood and HDPE composites (CAF-WPCs). X-ray photoelectron spectroscopy (XPS) and scanning electron microscopy (SEM) was used to investigate the chemical composition and morphology of the continuous aramid fiber before and after modification. The interfacial shear strength of CAF with WPCs was characterized by single fiber pull-out tests. Creep behavior of the resulting CAF-WPCs composites was also investigated.

## 2. Materials and Methods

### 2.1. Materials

Continuous aramid fibers (CAF) were provided from Sovetl Textile company, Dongguan, China. The average linear density of CAF was 1000D×3 and each 1000D fiber bundle contains 666 single fibers. Dopamine hydrochloride (DPA) and Vinyl triethoxysilane (VTES) were purchased from Macklin Biochemical company, Shanghai, China. Tris (hydroxymethyl aminomethane) was purchased from Zhanyun Chemical company, Shanghai, China. Iron (III) chloride (FeCl_3_), analytical reagent grade, was provided from Research Institute of Tianjin Guangfu Fine Chemical. Other chemicals (ethanol, water, and hydrochloric acid) were commercially available and used as received.

HDPE (5000S) pellets were provided by Daqing Petrochemical company, China. The melt index of this material is 0.7 g·10 min^−1^ (190 °C, 2.16 kg) and the density is 0.954 g·cm^−3^. Poplar wood veneers were ground into wood particles of 40–80 mesh using a grinder in the Lab. Maleic anhydride grafted polyethylene (MAPE) with the graft ratio of 0.9–1.2 wt% was purchased from Nantong Rizhisheng Polymer Materials company. Stearic acid (1801) and PE wax were commercially available in China.

### 2.2. Surface Modification of CAF

Before modification, continuous aramid fibers (CAF) were immersed in absolute ethanol for 48 h to remove the impurities on CAF surface and washed three times with deionized water and dried at 80 °C for use. After modification, the modified CAF was washed three times with deionized water and dried at 80 °C for use.

#### 2.2.1. VTES Treatment

For VTES treatment, 95% ethyl alcohol solution was used as the hydrolysis medium. VTES and 95% ethyl alcohol solution (1:50, *v*/*v*) were mixed for hydrolysis by stirring at 50 °C for 2 h. Next, 95% ethyl alcohol solution was added to the hydrolyzed solution until reaching a mixing ratio of VTES and 95% ethyl alcohol of 1:500 (*v*/*v*). CAF was immersed in the VTES-95% ethyl alcohol solution at room condition for 2 h (at a mass fraction ratio of VTES and CAF of 1:10).

#### 2.2.2. DPA Treatment

Modification of the aramid fibers with DPA for deposition of poly (dopamine) layers was carried out by immersion process, as described in the previous study [32]. First, dissolve Tris in distilled water to prepare 0.05 mol·L^−1^ Tris-HCl buffer with adding HCl to adjust the pH to 8.5. Then dissolve dopamine in the Tris-HCl buffer solution to a concentration of 2.0 g·L^−1^. Finally, immersed CAF in the dopamine solution prepared above and react at room temperature for 24 h.

#### 2.2.3. DPA and VTES Combined Treatment

For DPA and VTES modification, CAF was first modified by DPA treatment, and then excessive FeCl_3_ was Dissolved in VTES-95% ethyl alcohol solution to treat the dopamine modified fiber (CAFD) by immersing the fiber for 2 h (using a mass fraction ratio of VTES and CAF of 1:10).

In this study, CAF modified with VTES, DPA, and DPA and VTES were named as CAFV, CAFD, and CAFDV, respectively.

### 2.3. Preparation of CAF-Reinforced WPCs Composites

**Figure 1:**
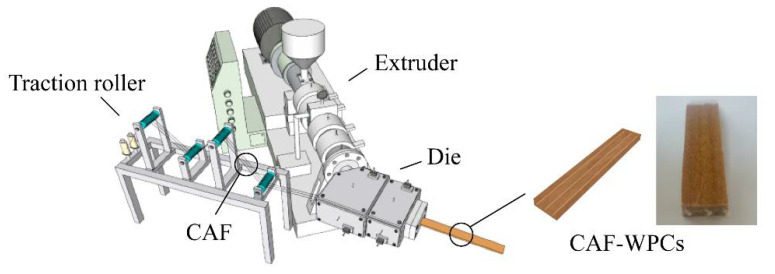
Schematic diagram of the processing and the shape of CAF reinforced WPCs.

The preparation of continuous fiber reinforced WPCs composites was described in our previous work [26,36]. Briefly, wood flour, HDPE, MAPE, and PE wax were mixed in the mixer with a certain ratio (Table 1) at ambient temperature. Using a co-rotating twin-screw extruder, the compounds were extruded and pulverized into pellets. Then, continuous fibers were drawn to the special die and extruded with the melt pellets together by a single screw extruder (Figure 1). The CAF reinforced WPCs composites were finally formed to a 4 × 50 mm^2^ (thickness × width) continuous plate and there were three bundles of aramid fibers in composites, with a mass fraction of 0.1%.

### 2.4. Characterization

#### 2.4.1. Scanning Electron Microscopy (SEM)

Frozen the CAF reinforced composites in liquid nitrogen for 5 min and breaking along the extrusion direction. Fibers and cryo-fractured surfaces were placed on the sample stage and sputter-coated with gold. The scanning electron microscopy (SEM, FEI company Quanta200, Hillsboro, TX, USA) was used to observe under a 15 kV accelerating voltage.

#### 2.4.2. X-ray Photoelectron Spectroscopy (XPS)

X-ray photoelectron spectroscopy (XPS, Thermo Fisher Scientific K-Alpha, Waltham, MA, USA) was used to analyze the surface element content of virgin and modified CAF. The samples were tested at 12 kV and 6 mA, the energy resolution is 0.5 eV.

#### 2.4.3. Mechanical Strength of CAF

The mechanical strength of CAF before and after modification was measured by a universal mechanical machine (CMT5540, MTS company, Eden Prairie, MN, USA) with the 100 mm·min^−1^ stretching speed according to GB/T14337-2008. Each group of samples was tested 10 times to determine the average values.

#### 2.4.4. CAF and WPCs Matrix Adhesion Measurement

**Figure 2:**
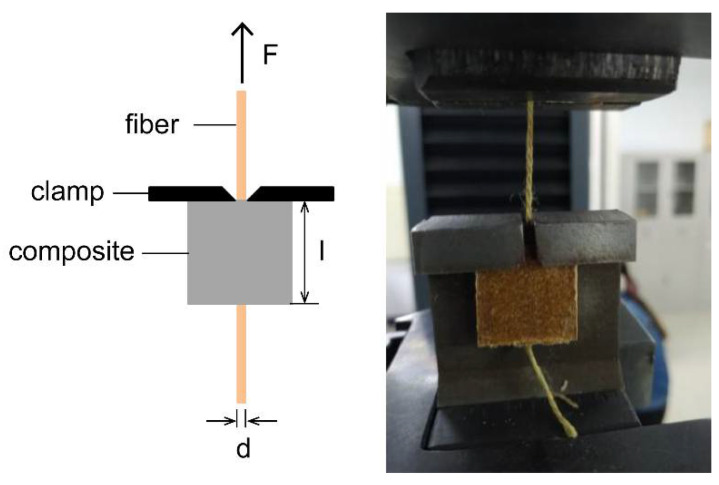
Illustration of the CAF reinforced WPCs pull-out experiment.

The single fiber pull-out test was used on a universal mechanical machine to measure the interfacial adhesion of CAF reinforced WPCs composites as illustrated by the schematic diagrams in Figure 2. The crosshead speed was 100 mm·min^−1^ and the interface shear strength (IFSS) τ calculation formula is as follows:

$$\tau =\frac{F}{\pi dl}$$

where *F* refers to the maximum force, *d* is the diameter of CAF, and *l* is the length of test composites. Each test was repeated 10 times to determine the average values.

#### 2.4.5. Mechanical Tests

The tensile and impact strength of CAF reinforced WPCs were tested using the universal testing machine and an impact tester (JC-5, Chengde, China) according to ASTM D638-03 and a Chinese standard of GB/T 1843.1-2008, respectively. In the tensile test, the sample is dumbbell-shaped with the size of 165 × 20 × 4 mm^3^ (length × width × thickness), and the width at the narrow part is 12.7 mm, the tensile speed is 5 mm·min^−1^. In the impact test, the sample is rectangular with the size of 80 × 10 × 4 mm^3^ (length × width × thickness). Test carried out with unnotched Izod impact tests at a speed of 3.8 m·s^−1^ while the impact energy was 2 J. Each group of samples was tested 5 times to determine the average values.

#### 2.4.6. Creep Behavior Analysis

The short-term creep properties of CAF reinforced WPCs composites were tested using a rotational rheometer (AR2000ex, TA Instruments, New Castle, DE, USA). The samples were 32 × 10 × 3 mm^3^ (length × width × thickness). The test was carried out at 60 °C with an application of 2 MPa stress, and 30 min for loading and releasing, respectively.

## 3. Results and Discussion

**Figure 3:**
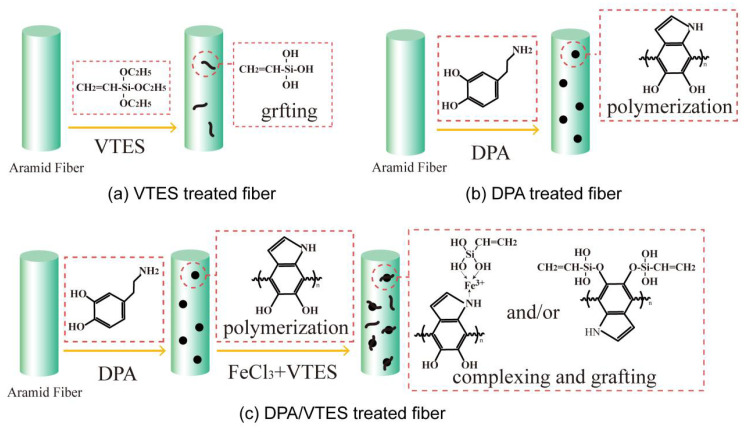
Procedures for CAF modification: (**a**) vinyl triethoxysilane-treated, (**b**) dopamine-treated, and (**c**) dopamine and vinyl triethoxysilane-treated.

**Figure 4:**
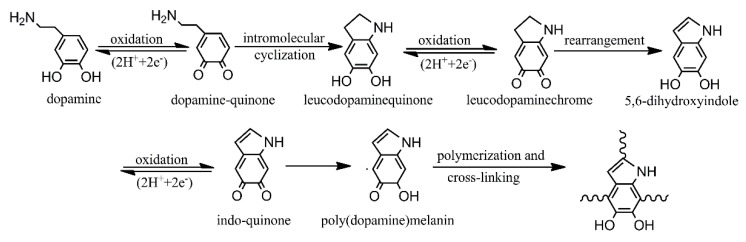
Potential polymerization mechanism of dopamine [32].

The reaction mechanisms of VTES treatment, DPA treatment, and DPA and VTES treatment on CAF are described in Figure 3. For VTES treatment, the silane VTES is hydrolyzed to generated silanol, which can react with the carboxyl groups on the CAF surface through esterification (Figure 3a). For DPA treatment, the polydopamine polymer is deposited on the CAF surface by monomer oxidative and self-polymerization (Figure 3b). In this process, dopamine is first oxidized to dopamine-quinone, and then the product undergoes intramolecular cyclization. After a series of oxidation or rearrangement reactions, the final monomers can branch react to each other. Finally, a thin layer of PDA forms on the aramid fiber through self-assembly polymerization and cross-linking (Figure 4). Lee et al. performed a single-molecule study on the substrate and adhesive properties of mussel though the reaction mechanism between the polydopamine polymer and substrate remains unclear. According to their research, the oxidation of DPA formed high-strength irreversible covalent bonding on the CAF surface [28]. For DPA and VTES combined treatment, polydopamine deposited on CAF surface and introduced active -OH groups that can react with silane coupling agent VTES. Further, Fe^3+^ with six coordination sites that can bond with the -NH_2_ groups on the surface of poly (dopamine) deposited aramid fiber [37]. The coordinatively unsaturated sites of Fe^3+^ provide active sites to the poly (dopamine) deposited fiber and promote additional VTES grafting onto the fiber surface. (Figure 3c).

### 3.1. Surface Morphology of CAF

**Figure 5:**
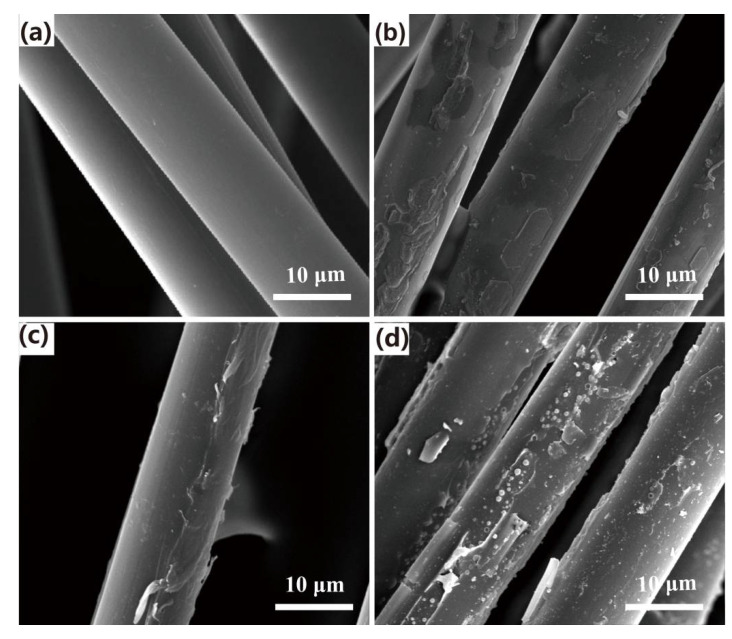
SEM pictures of CAF: (**a**) virgin, (**b**) dopamine-treated, (**c**) vinyl triethoxysilane-treated, and (**d**) dopamine and vinyl triethoxysilane-treated.

SEM analysis was performed to determine the detailed micromorphology of the CAF. The pristine aramid fibers displayed a smooth and uniform surface after extraction by ethanol solvent (Figure 5a). After dopamine treatment, the surface morphology of the fibers became rougher due to the poly (dopamine) deposition, caused by dopamine oxidative self-polymerization (Figure 5b). Similarly, the fiber surface after VTES treatment appeared rough due to the grafting of the silane coupling agent (Figure 5c). Compared with individual treatment with either pure DPA or VTES, there was obviously increased chemical deposition on the surface of fiber increased for DPA and VTES combined treatment, this result indicated that the presence of dopamine effectively promoted the grafting of VTES onto the CAF. Fiber modification resulting in larger surface area and reactivity, which may increase the interfacial adhesion between CAF and WPCs matrix.

### 3.2. Surface Chemical Composition of CAF

**Figure 6:**
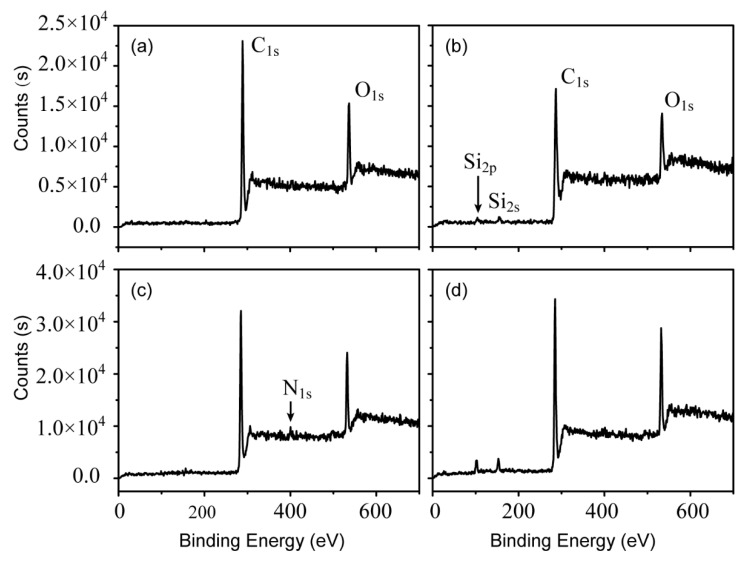
XPS spectra of aramid continuous fiber: (**a**) original, (**b**) vinyl triethoxysilane- treated, (**c**) dopamine-treated, and (**d**) dopamine and vinyl triethoxysilane-treated.

The XPS spectra of the surface of pristine and modified CAF were used to verify the mechanism of surface modification on CAF (Figure 6). The XPS spectroscopy showed the C_1s_, N_1s_, and O_1s_ peaks at 289, 402, 536 eV, respectively, in both pristine CAF and modified CAF, indicating that C, N, and O elements are present in CAF [18]. Spectroscopy of the VTES-treated aramid fiber surface showed the characteristic peaks in the region of 151–155 eV assigned to Si_2s_ and 100–104 eV assigned to Si_2p_, respectively. This result indicated that VTES was successfully grafted on the CAF, which is consistent with the SEM results. Relative atomic percentages of O and N on the CAF increased after DPA modification (Table 2), and the O and C and N and C ratios of the CAF surface increased from 21.6 and 2.8 (virgin fiber) to 23.0 and 6.1 due to modification. These increases can be explained by the introduction of -NH_2_ and -OH groups on the fiber surface after polydopamine deposition [31]. Fe element also appeared on fiber treated with the combination of DPA and VTES due to the successful complexation of poly (dopamine) deposited fiber and VTES (Figure 6d). After complexation on fiber, the coordinatively unsaturated sites of Fe^3+^ provided additional active sites on the modified fiber for the grafting of VTES [37]. Therefore, the relative atomic percentages of silicon and O and C ratio of fiber surface increased from 3.3 and 21.6 to 5.75 and 26.5, respectively. Overall, dopamine promoted the grafting reaction of fiber and coupling agent in the presence of Fe^3+^.

### 3.3. Interfacial Shear Strength of CAF

**Figure 7:**
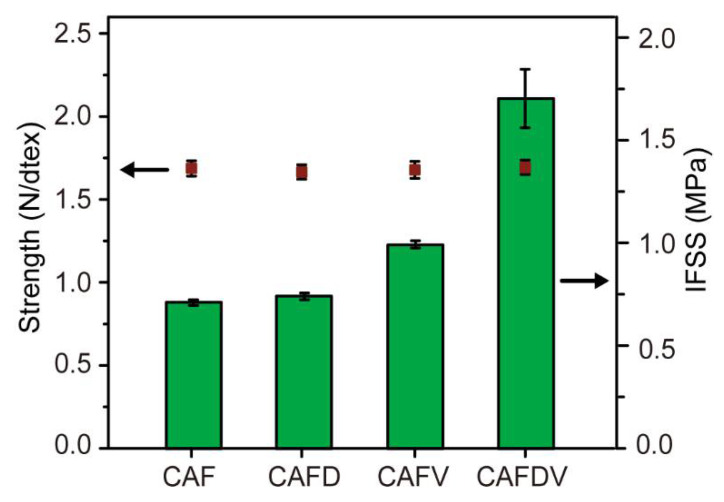
Mechanical strength and IFSS of the CAF.

The interface shear strength (IFSS) between CAF and WPCs was characterized using a single-fiber pull-out test, and the results are shown in Figure 7. The IFSS value of the prepared CAFD-WPCs was 0.74 MPa, 4.2% higher than that of the CAF-WPC composite (0.71 MPa). This increased IFSS can be attributed to the introduction of polar groups on the polydopamine deposited CAF. The -NH_2_ and -OH groups confer high affinity to the hydroxyl groups of matrixes. However, the slight improvement suggests that the number of hydrogen bonds fiber exposed is limited, so does not sufficiently contribute to interface bonding in the composites. Treatment with VTES alone increased the IFSS of the resulting composite from 0.71 to 0.99 MPa, 39% higher than that of the untreated system. The use of VTES improved the surface polarity of the inert CAF, which helps to improve interface compatibility in composites. Using both polydopamine and VTES modification on fiber increased the interfacial strength (IFSS) of the resulting composites significantly. The tested CAFDV-WPC composite exhibited the highest IFSS value of 1.7 MPa, 139.4% higher than that of pristine fiber. In the presence of Fe^3+^, poly (dopamine) promoted the grafting of more coupling agents onto the fiber surface, resulting in greatly improved interfacial compatibility between phases. In this system, the polar hydroxyl and amino groups on CAF can extensively form hydrogen bonds with the wood flour. Additionally, the vinyl groups of coupling agent VTES can generate free radicals and react with the HDPE backbone during the extrusion process [18]. As a result, a good interface can be formed between CAF and the WPCs in the final composites.

The tensile strength of virgin continuous fiber was measured as 1.7 N·dtex^−1^, and the strength of fiber was basically unchanged after modification (Figure 7), which indicating this modification has no negative effect on fiber strength. This is because both dopamine deposition and VTES grafting reaction only occur on the CAF surface, and does not break the molecular chain structure of continuous aramid fiber.

### 3.4. Fracture Morphology Analysis of Composites

**Figure 8:**
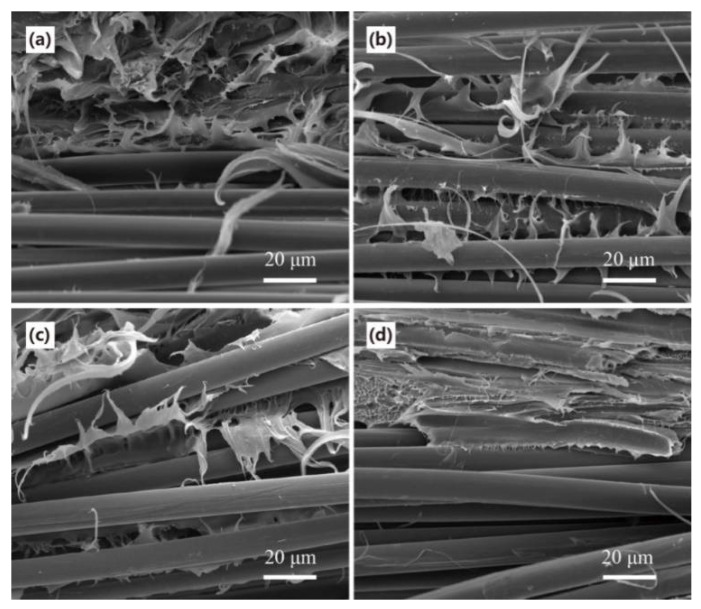
Fracture morphology of the CAF reinforced WPCs: (**a**) virgin, (**b**) dopamine-treated, (**c**) vinyl triethoxysilane-treated, and (**d**) dopamine and vinyl triethoxysilane-treated.

Structural morphology characteristics of the prepared continuous aramid fiber reinforced WPCs were investigated to assess the interfacial bonding between CAF and WPCs. For virgin CAF reinforced WPCs, the fracture surface appeared smooth with almost no matrix adhesion, indicating weak interfacial bonding between CAF and WPCs (Figure 8a). Compared to materials modified by either dopamine or VTES, adherents were observed on the surface of the fiber, and the wire drawing phenomenon existed in the fracture composites, indicating that the interface was improved. Stronger interface adhesion and more obvious drawing phenomenon were observed in composites reinforced using DPA and VTES modified fibers (Figure 8d). The results indicated increased interface compatibility between the modified CAF and WPCs, increasing interfacial adhesion strength (Figure 7).

### 3.5. Mechanical Properties of Composites

**Figure 9:**
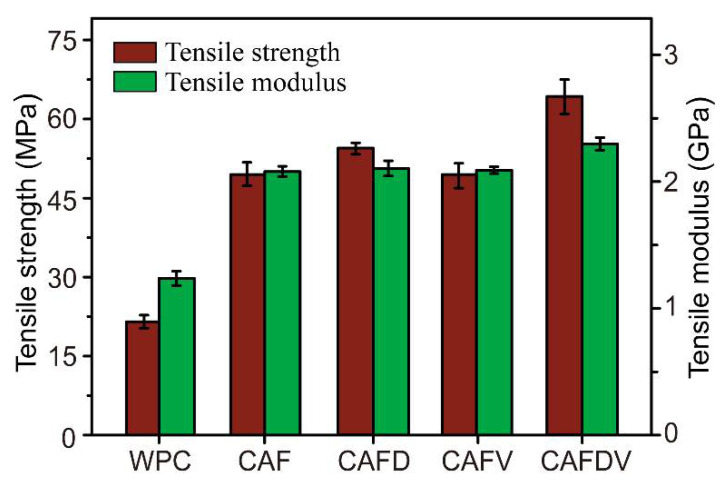
Tensile properties of the CAF reinforced WPCs.

Adding CAF significantly improved the mechanical performance of the resulting composites. Compared with pure WPCs, the tensile strength and modulus of CAF-reinforced WPCs increased by 130% and 68%, respectively (Figure 9). The tensile stress loaded on the composites can be transferred from the WPCs to CAF through interfacial shear. The continuous aramid fibers withstand most of the stress, resulting in the enhancement in strength and modulus. As the load increases, the cracks were generated and propagated in the WPCs matrix and the interface between CAF and WPCs, which ultimately destroyed the composites [26]. In this system, when the external force exceeds the interface bonding strength between CAF and WPCs matrix, the fiber pulls out of the matrix. At this time, the high-strength and modulus CAF subjected less stress than their own tensile strength. Therefore, the improved interfacial property is a major contributor to the overall strength of the composites. After modification, treated fiber improved tensile strength and modulus to a greater extent than virgin fiber because of the higher stress transfer efficiency between CAF and WPCs. Dopamine treated CAF introduced -NH_2_ and -OH groups on the fiber surface with high affinity for the hydroxyl groups of the matrix. The use of VTES improves the surface polarity of the inert CAF. However, the number of polar groups on CAFD and CAFV is limited, therefore insufficient to promote interfacial adhesion in the composite and resulting in a slight enhancement in tensile strength. For DPA and VTES combined treatment (CAFDV), polydopamine deposited on CAF surface and introduced active -OH groups that can react with silane coupling agent VTES. Further, in the presence of Fe^3+^, the -NH_2_ groups on the CAF can be complexed with VTES, thereby promote additional VTES grafting onto the fiber surface. Strong interfacial combination between CAFDV and WPC matrix significantly increased tensile strength of the resulting composites. The tensile strength and modulus of CAFDV-WPCs were 64.2 MPa and 2.3 GPa, which are 29 and 10% higher than that of untreated fiber (49.6 MPa and 2.1 GPa). Combined with the analysis of IFSS results, modification resulted in improved interface bonding, allowing CAF to withstand greater loads through interfacial shear.

**Figure 10:**
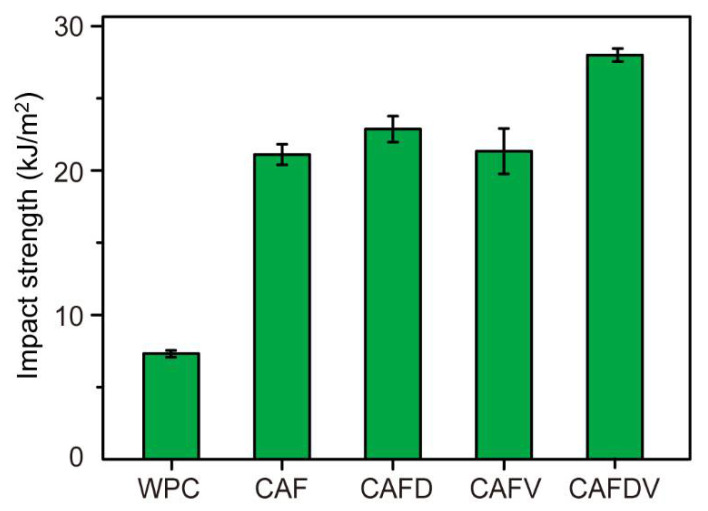
Impact strength of the CAF reinforced WPCs.

Compared with pure WPCs, the impact strength of CAF reinforced WPCs was increased by 283% maximally, which is difficult to achieve with common reinforcement (Figure 10). Compared with virgin CAF, DPA and VTES-treated CAF further increased the impact strength of CAF reinforced WPCs composites from 21.1 kJ·m^−2^ to 28.0 kJ·m^−2^, an increase of 32.7%. This is due to the enhanced interface compatibility. Improved interfacial bonding allowed the impact loading transferred steadily from the WPCs matrix to the fibers, which reduced crack generation and propagation at the interface between CAF and WPCs or in the matrix. This process absorbed most of the impact energy, resulting in improved impact strength. Besides, the enhancement of CAF on the impact strength of CAF-reinforced composites is more obvious than tensile strength. This is because unlike the tensile stress, the direction of impact stress is perpendicular to the fiber direction in composites. The CAF restricts the movement of HDPE molecular chains, thus the stress dissipation in composites is confined [26]. This allowed the composites to withstand greater loads, finally resulting in improved impact strength.

Interestingly, the effect of fiber modification on the interfacial shear strength of the composites is more obvious than that of mechanical properties. These results may be caused by the use of different test samples in IFSS and mechanical tests. There was a larger fiber volume fraction in the mechanical test sample (three bundles) than that in the IFSS sample (one bundle) and this may affect the formation of interface defects in composites. Cracks at the interface promote the debonding of CAF from the WPCs composites under external force. Another difference between the samples is that the samples used in the IFSS test were prepared by hot pressing method. Compared to the extrusion process, this method included an increased matrix melting time and additional pressure during the composite preparation process to fully impregnate the continuous fiber into the WPCs matrix.

### 3.6. Creep Property of Composites

**Figure 11:**
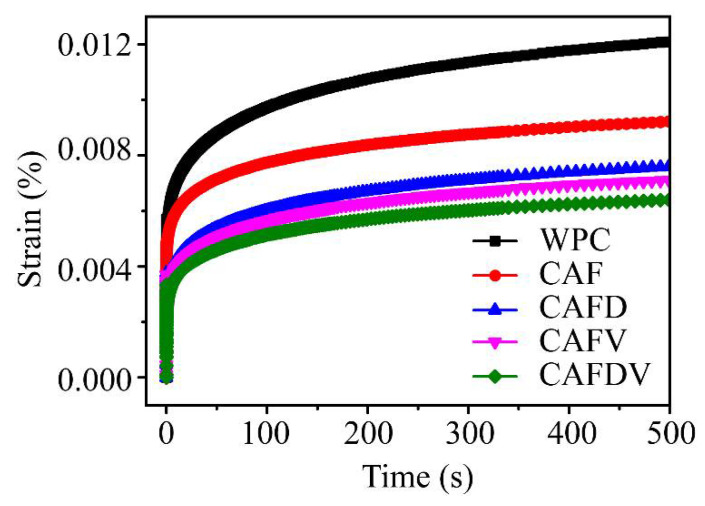
Creep curves of the CAF-reinforced WPCs.

The short-term creep performance of composites was usually used to characterize the creep resistance and the interface compatibility of the composites [5]. The creep mechanism at molecular level for unmodified and modified CAF reinforced WPCs is mainly due to the movement and deformation of the HDPE molecular chain and the deformation of the material caused by the slippage of the polymer molecular chain at the interface. The addition of aramid fiber effectively improved the creep resistance of the composite (Figure 11). CAF reduced the creep deformation of composite materials regardless of with modification or not. This is due to the high dimensionally stable CAF restricted the movement of PE molecular chains. Besides, compared to non-modified fiber, treated fiber-reinforced WF and HDPE composites exhibited smaller creep due to the enhanced interfacial bonding between CAF and WPCs. The improved interface provided a stronger interfacial shear effect for restraining the motion of HDPE polymer molecules at the interface. Composites reinforced with DPA and VTES treated fiber exhibited the highest creep resistance among all fiber-reinforced WPC composites, indicating better interfacial compatibility in composites.

## 4. Conclusions

This study provided an effective and nondestructive functionalization method for continuous aramid fiber to develop high-performance continuous fiber-reinforced WPCs. Dopamine and functional silane VTES were successfully deposited and grafted on the fiber surface as evidenced by XPS and SEM analysis. The interfacial adhesion between CAF and WPCs matrix was improved and the adhesion strength (IFSS) for materials prepared with modified CAF increased by 143%. Additionally, the tensile strength, tensile modulus, and impact strength of the modified CAF reinforced WPCs were further enhanced due to stronger interface shear force in the composites. The results indicate that suitable interfacial adhesion between components is the prerequisite to fully utilize the reinforcing ability of CAF, and strong interface bonding is critical for composites with improved properties.

## Figures and Tables

**Table 1:** Formulation of the composites for extrusion.

	WF	HDPE	MAPE	PE Wax	Stearic Acid
Content (wt%)	50	44	4	1	1

**Table 2:** Surface elemental composition of CAF.

Sample	Atomic Percent (%)					Atomic Ratio	
	C	O	N	Si	Fe	O/C	N/C
CAF	80.01	17.32	2.67	-	-	21.6	2.8
CAFD	77.44	17.80	4.76	-	-	23.0	6.1
CAFV	75.72	16.13	4.86	3.3		21.3	6.4
CAFDV	70.99	18.78	2.84	5.75	1.63	26.5	4

## Data Availability

All data included in this study are available upon request by contact with the corresponding author.
